# The impact of free vaccination policies under the Korean Influenza National Immunization Program: Trends in influenza vaccination rates in South Korea from 2010 to 2019

**DOI:** 10.1371/journal.pone.0262594

**Published:** 2022-01-20

**Authors:** Jeongmin Seo, Juwon Lim

**Affiliations:** 1 Department of Internal Medicine, Seoul National University Hospital, Seoul, Korea; 2 International Healthcare Center, Seoul National University Hospital, Seoul, Korea; Seoul National University College of Medicine, REPUBLIC OF KOREA

## Abstract

**Background:**

Annual vaccination for influenza is recommended for high-risk populations for its high morbidity and mortality. South Korea provides free influenza vaccination to some target groups under the National Immunization Program (NIP), and discrepantly high vaccination rates are observed in such populations. In this study, we analyzed the trends in influenza vaccination rates and evaluated the impact of the recent expansion of financial coverage to children ≤12 years and pregnant women.

**Methods:**

We conducted a cross-sectional study with nationwide survey data from Korea National Health and Nutrition Examination Survey (KNHANES). From 2010 to 2019, we evaluated the trends in influenza vaccination rates of the following four target groups: children ≤12 years, adults ≥65 years, pregnant women, and people with chronic diseases.

**Results:**

In total, 80,861 individuals were analyzed. From 2017 to 2019, the vaccination coverage of children ≤12 years increased from 66.2% to 83.1%; pregnant women from 44.1% to 68.5% (comparing the mean of 2010–2017 and 2018–2019, P <0.001 for both). The elderly ≥65 years showed the highest rates (85.8% in 2019), while people with chronic diseases marked the lowest (41.9% in 2019). People with liver diseases showed the lowest vaccination rate of 27.8%, while that of other common diseases ranged between 31.7–44.1%.

**Conclusion:**

The discrepancy between target groups corresponds to their financial coverage under NIP. The recent expansion of financial aids to children ≤12 years and pregnant women was followed by significant increases in vaccination rates in both groups. We suggest that free vaccination policy is one of the most effective strategies to enhance vaccination coverage, and we call for its expansion to other under-vaccinated target groups, especially people with chronic diseases.

## Introduction

Influenza is one of the leading causes of morbidity and mortality, infecting 5–10% of adults and 20–30% of children worldwide [1]. In South Korea, 10–20% of adults are infected with seasonal influenza each year. It is estimated that influenza and its complications lead to 43,000 hospitalizations and 2900–5300 deaths per year, costing 125 million US dollars in the adult population [2–6].

Influenza can affect all individuals, but the risk of complications and deaths varies greatly. High-risk groups include children aged <5 years, adults aged ≥50 years, pregnant women, and people with chronic diseases [7,8]. Annual vaccination is a well-established prevention method. In order to make the most of the limited resources, many countries focus on high-risk groups when setting policies to encourage vaccination in high-risk groups. In 2003, World Health Organization (WHO) set the vaccination goal for high-risk populations as 75% [9]. In the USA, Healthy People 2030 initiative has set the goal of 70% for all population ≥6 months of age [10]. However, many countries fail to meet this standard [11]. South Korea has comparatively high vaccination rates, but significant disparities among the target groups have been reported. The vaccination rates of the elderly amount to 80%, while those of people with chronic diseases remain around 30% [12].

To interpret this discrepancy, it is important to address the role of the National Immunization Program (NIP) of South Korea. NIP for influenza was implemented in 1997 targeting the elderly of low socioeconomic status [8]. Since 2005, the elderly aged ≥65 years have received influenza vaccinations free of charge. The financial coverage to the elderly was considered as the key factor for their high vaccination rates. Our previous study has revealed that high household income was significantly associated with high vaccination coverage in all target groups, while this correlation selectively disappeared in the population who were granted free vaccinations [12]. It clearly demonstrates the impact of free vaccination policies which successfully removed the financial barrier and improved the vaccination rates significantly.

In recent years, NIP expanded its financial coverage to other target groups. Free vaccinations have been provided to children aged 6 months to 5 years since 2017, children aged ≤12 years since 2018, and pregnant women since 2019 [8]. The number of financially aided individuals amounted to 13 million in 2018, a number representing 26% of the total population [13].

This study aims to evaluate the impact of recent alterations of vaccination policies regarding children and pregnant women in South Korea. By evaluating the trends of influenza vaccination rates from 2010 to 2019 in different target groups, we analyzed the impact and limitations of current vaccination policies. By doing so, we aim to address the next under-represented target group to focus on and to provide suggestions for the improvement of policies.

## Materials and methods

### Study design and population

We analyzed the data retrieved from the Korea National Health and Nutrition Examination Survey (KNHANES) conducted by Korea Disease Control and Prevention Agency (KDCA). KNHANES is an annual population-based cross-sectional survey to assess the health and nutritional state of the Korean population. About 10,000 individuals aged 1 year and older participate in the survey each year. It comprises three components: health examination, health interview, and nutrition survey [14,15].

We collected 10-year data from 2010 to 2019. The response rate to the survey ranged between 74.7% and 80.8% [16].

### Measures

The comparison of high-risk groups designated by Center for Disease Control and Prevention (CDC) and KDCA is presented in S1 Table [7,8]. Four target groups were selected based on these national recommendations: children, the elderly, pregnant women, and people with chronic diseases. According to KDCA, children aged 6–59 months, children and adolescents aged 5–18 years living in close proximities, and adults aged ≥50 years are at high risk of complications. To assess the impact of recent policy alterations, we applied the age cutoffs of financial aids instead: children aged ≤12 years and adults aged ≥65 years. Women who reported being pregnant at the time of the survey were classified as pregnant women.

Chronic diseases were classified into the following categories: malignancies (stomach, liver, colon, breast, cervical, lung, thyroid, and others), diabetes mellitus, kidney diseases (chronic renal disease), heart diseases (coronary heart disease, myocardial infarction, angina, and stroke), lung diseases (bronchial asthma, tuberculosis) and liver diseases (chronic viral hepatitis and liver cirrhosis) [17].

Self-reported influenza vaccination status was obtained from health interviews by asking the receipt of influenza vaccines in the past 12 months. The vaccination rate was calculated as the number of individuals who responded ‘Yes’ divided by the sum of individuals with ‘Yes’, ‘No’, ‘Unknown’, and ‘null’.

The following sociodemographic factors were examined: gender (male or female), residency area (city or rural), level of education (≤9, 10–12, ≥13 years), and household incomes (in quartiles).

### Statistical analysis

Bivariate associations and time trends of categorical variables were assessed using the chi-square test. Associations between sociodemographic factors and vaccination rates were evaluated using multivariate logistic regression. Two-tailed P-values of <0.05 were considered statistically significant. P-value, P-for-trend, 95% confidence interval (CI), and adjusted odds ratio (aOR) were reported as indicated. The sample weights were adjusted accounting for selection probabilities, survey nonresponse, and post-stratification [18]. These data were analyzed using STATA® version 17 for Windows (StataCorp LLC, College Station, TX).

## Results

### Demographic characteristics and Influenza vaccination rates

Table 1 illustrates the general characteristics of the study population. In total, 80,861 individuals were analyzed. Among them, 45.5% were male and 81.2% lived in the city. Target groups accounted for 43.8% of the total population: children aged ≤12 years, 14.8%; the elderly aged ≥65 years, 19.7%; pregnant women, 0.4%; people with chronic diseases, 8.9%. The types of chronic diseases in order of prevalence were as follows: lung diseases (3.3%), diabetes mellitus (2.8%), malignancies (1.7%), heart diseases (1.1%), liver diseases (0.9%), and kidney diseases (0.2%). The individuals with two or more diseases were counted more than once.

**Table 1:** General characteristics and influenza vaccination rates (n = 80,861).

				Total		Vaccinated		P-value
Sociodemographic factors				n	%	n	%	
	Age (years)		≤12	11,944	14.8	6,773	56.7	<0.001
			13–64	52,975	65.5	12,350	23.3	
			≥65	15,942	19.7	10,690	67.1	
	Gender		Male	36,827	45.5	12,606	34.2	<0.001
			Female	44,034	54.5	17,207	39.1	
	Region		City	65,626	81.2	23,542	35.9	<0.001
			Rural	15,235	18.8	6,271	41.2	
	Education		≤9 years	21,056	33.7	11,528	54.7	<0.001
			10–12 years	17,968	28.8	5,430	30.2	
			≥13 years	23,463	37.5	6,336	27.0	
	Income		Lowest quartile	19,955	24.9	7,160	35.9	0.131
			Second quartile	20,096	25.1	7,415	36.9	
			Third quartile	20,140	25.1	7,488	37.2	
			Highest quartile	20,003	24.9	7,563	37.8	
Non-target group								
	Age 13–64, no diseases, not pregnant			45,477	56.2	9,793	21.5	<0.001
Target group				35,384	43.8	20,020	56.6	
	Age (years)		≤12	11,944	14.8	6,773	56.7	<0.001
			≥65	15,942	19.7	10,690	67.1	
	Pregnant women			291	0.4	104	35.7	
	Age 13–64 with chronic diseases			7,210	8.9	2,455	34.0	
		Disease type	Malignancies	1,377	1.7	548	39.8	<0.001
			Diabetes mellitus	2,292	2.8	866	37.8	
			Kidney diseases	143	0.2	63	44.1	
			Heart diseases	920	1.1	336	36.5	
			Lung diseases	2,667	3.3	845	31.7	
			Liver diseases	734	0.9	204	27.8	

*Heart diseases: Coronary heart disease, myocardial infarction, angina, stroke.

*Kidney diseases: Chronic renal disease.

*Lung diseases: Bronchial asthma, tuberculosis.

*Liver diseases: Chronic viral hepatitis, liver cirrhosis.

Higher vaccination rates were significantly related to the following sociodemographic factors: age ≤12 and age ≥65, female gender, rural residency, and less educational level. Household income was not related to vaccination rates.

### The association between sociodemographic factors and influenza vaccination rates in each target group

Table 2 evaluates the association between sociodemographic factors and influenza vaccination rates in the four selected target groups. The factors related to higher vaccination rates in each target group were as follows: in age ≤12, female gender and less educational level; age ≥65, female gender; pregnant women, none; people with chronic diseases, female gender, rural residence, and low educational level. Household income was not associated with vaccination rates in all target groups.

**Table 2:** Influenza vaccination rates and sociodemographic factors in target groups.

			Age ≤ 12					Pregnant women					Age 13–64 with chronic diseases					Age ≥ 65				
			Total		Vaccinated		P-value	Total		Vaccinated		P-value	Total		Vaccinated		P-value	Total		Vaccinated		P-value
			11,944		6,773			291		102			7,210		2,455			15,942		10,690		
Sociodemographic factors			n	%	n	%		n	%	n	%		n	%	n	%		n	%	n	%	
	Gender																					
		Male	6,230	52.2	3,463	55.6	0.010						3,438	47.7	1,009	29.3	<0.001	6,859	43.0	4,510	65.8	<0.001
		Female	5,714	47.8	3,310	57.9		291	100	102	35.1		3,772	52.3	1,446	38.3		9,083	57.0	6,180	68.0	
	Region																					
		City	10,070	84.3	5,750	57.1	0.044	241	82.8	87	36.1	0.746	5,823	80.8	1,934	33.2	0.002	11,231	70.4	7,565	67.4	0.933
		Rural	1,874	15.7	1,023	54.6		50	17.2	15	30.0		1,387	19.2	521	37.6		4,711	29.6	3,125	66.3	
	Education																					
		None	11,944	100.0	6,405	53.6	<0.001															
		≤9 years	896	7.5	368	41.1		5	1.8	2	40.0	0.391	2,230	30.9	955	42.8	<0.001	10,358	73.3	7,746	74.8	0.058
		10–12 years						57	21.0	17	29.8		2,425	33.6	784	32.3		2,349	16.6	1,782	75.9	
		≥13 years						209	77.1	83	39.7		2,486	34.5	707	28.4		1,433	10.1	1,069	74.6	
	Income																					
		Lowest quartile	2,933	24.6	1804	61.5	0.027	53	18.3	22	41.5	0.056	1,952	27.1	696	35.7	0.306	3,955	25.1	2,602	65.8	0.481
		Second quartile	2,979	24.9	1929	64.8		82	28.4	19	23.2		1,792	24.9	596	33.3		3,955	25.1	2,692	68.1	
		Third quartile	2,989	25.0	1913	64.0		82	28.4	32	39.0		1,725	23.9	569	33.0		3,955	25.1	2,665	67.4	
		Highest quartile	2,961	24.8	1850	62.5		72	24.9	28	38.9		1,700	23.6	580	34.1		3,898	24.7	2,653	68.1	

### Trends of vaccination rates among the target groups

**Fig 1:**
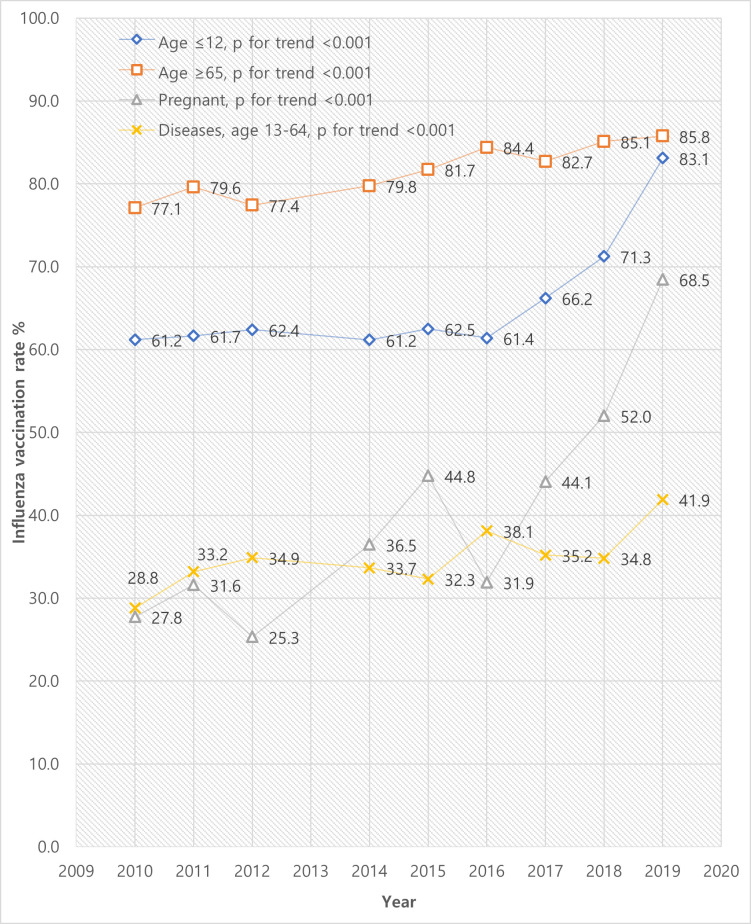
Trends of influenza vaccination rates in target groups during 2010–2019.

The trends of influenza vaccination rates among the target groups are demonstrated in Fig 1. The elderly aged ≥65 years showed the highest rates throughout the study period, amounting to 85.8% in 2019. In contrast, people with chronic diseases remained low with little increase in vaccination rates. The other two groups showed significant increase in the last 2 years. From 2017 to 2019, the vaccination rates of children aged ≤12 years increased from 66.2% to 83.1%, and those of pregnant women increased from 44.1% to 68.5%.

**Fig 2:**
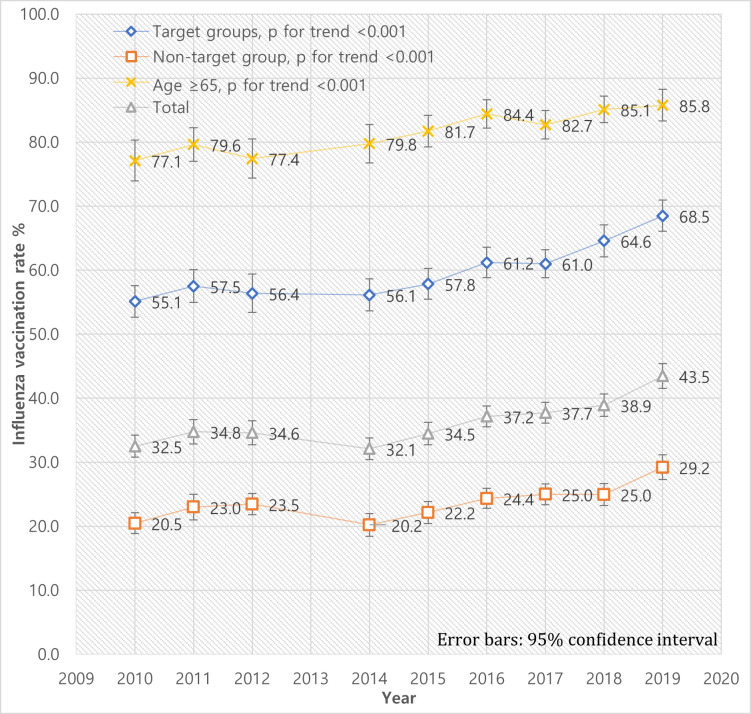
Trends of influenza vaccination rates between composite target groups and non-target group during 2010–2019.

Fig 2 shows the composite vaccination rates of the four target groups designated above. The vaccination rates of target groups amounted to 68.5% in 2019, while those of non-target group marked 29.2%. The discrepancy existed consistently throughout the study period. The trend of the elderly is plotted together to illustrate to which extent it is driving the vaccination rates of the target groups.

**Fig 3:**
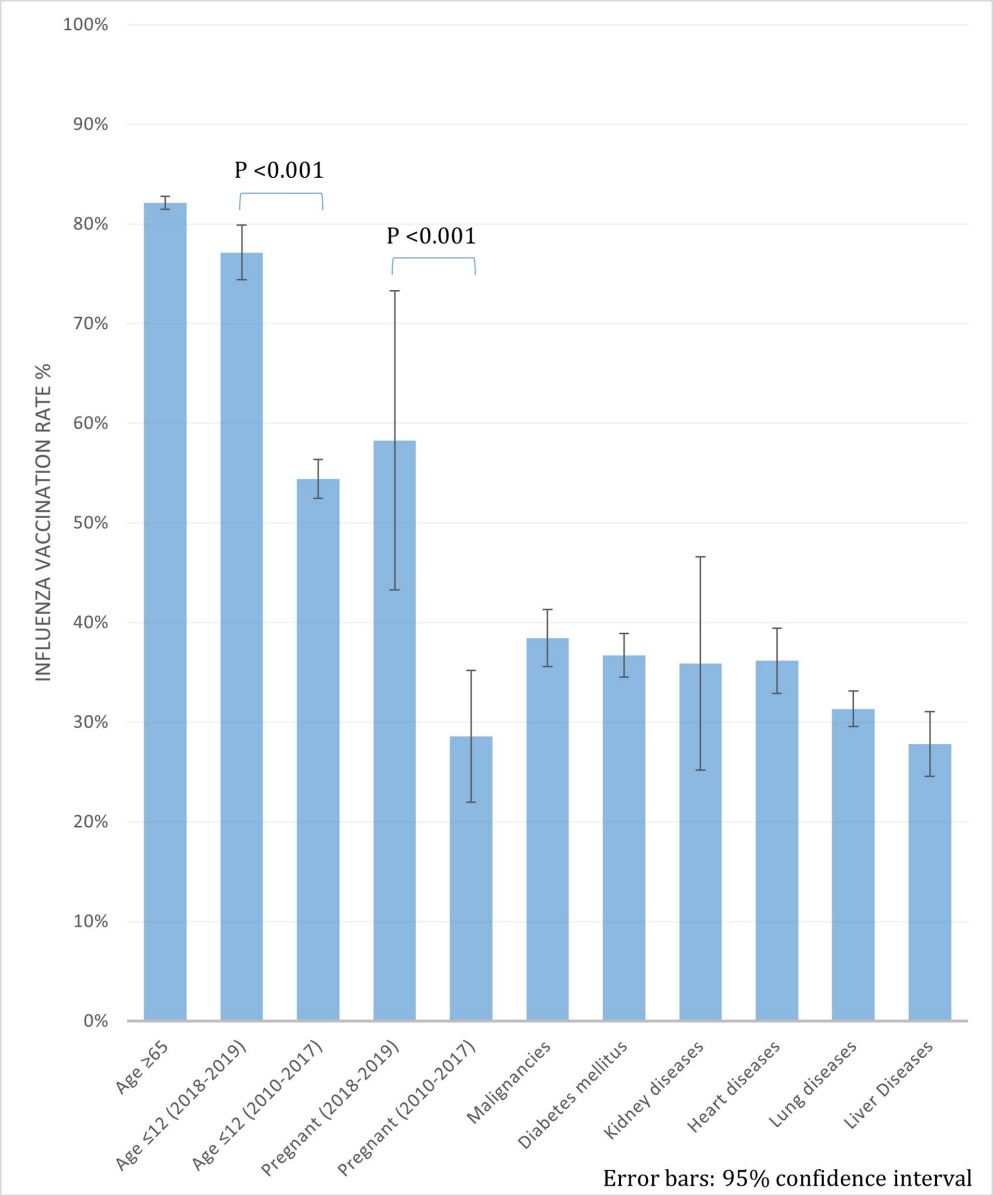
The comparison of influenza vaccination rates according to different periods and the types of chronic diseases.

Fig 3 compares this increase: between 2010–2017 and 2018–2019, there are statistically significant increases in vaccination rates in children aged ≤12 years and pregnant women (P <0.001 for both).

### Vaccination rates of people with chronic diseases

The vaccination rates differed significantly according to the type of chronic disease. In Table 1, the vaccination rates of the people with liver diseases marked the lowest of 27.8%, while those of other common diseases ranged between 31.7–44.1%. Table 3 evaluates the association between sociodemographic factors and vaccination rates in people with chronic diseases. The following factors were significantly related to higher vaccination coverage in people with chronic diseases: female gender, aOR 1.39; less education, aOR 1.70. The type of disease also showed significant correlations. Compared to liver diseases, other diseases showed significantly higher vaccination rates: malignancies, aOR 1.45; diabetes mellitus, 1.43; kidney diseases, 1.37; heart diseases, 1.29; lung diseases, 1.15. The vaccination rates according to the type of diseases are illustrated in Fig 3.

**Table 3:** Influenza vaccination rates according to sociodemographic factors in people with chronic diseases (age 13–64).

			Total		Vaccinated		P-value	aOR	95% CI	P-value
Sociodemographic factors			n	%	n	%				
	Gender	Male	3,438	47.7	1,009	29.3	<0.001	1		<0.001
		Female	3,772	52.3	1,446	38.3		1.39	(1.25–1.54)	
	Region	City	5,823	80.8	1,934	33.2	0.001	1		0.073
		Rural	1,387	19.2	521	37.6		1.12	(0.99–1.27)	
	Education	≤9 years	2,230	30.9	955	42.8	<0.001	1.70	(1.52–1.98)	<0.001
		10–12 years	2,425	33.6	784	32.3		1.15	(1.01–1.30)	0.030
		≥13 years	2,486	34.5	707	28.4		1		
	Income	Lowest quartile	1,952	27.1	696	35.7	0.430	1		
		Second quartile	1,792	24.9	596	33.3		0.95	(0.83–1.09)	0.450
		Third quartile	1,725	23.9	569	33.0		0.98	(0.85–1.12)	0.738
		Highest quartile	1,700	23.6	580	34.1		1.12	(0.97–1.30)	0.118
Disease type		Malignancies	1,377	19.1	548	39.8	<0.001	1.45	(1.18–1.78)	<0.001
		Diabetes mellitus	2,292	31.8	866	37.8		1.43	(1.18–1.73)	<0.001
		Kidney diseases	143	2.0	63	44.1		1.37	(0.84–2.26)	0.21
		Heart diseases	920	12.8	336	36.5		1.29	(1.04–1.62)	0.020
		Lung diseases	2,667	37.0	845	31.7		1.15	(0.96–1.39)	0.126
		Liver diseases	734	10.2	204	27.8		1		

*aOR: Adjusted odds ratio; 95% CI: 95% confidence interval.

## Discussion

In this cross-sectional study using national survey data, we examined the trends of influenza vaccination rates from 2010 to 2019 in each target group. In total, influenza vaccination rates in South Korea steadily increased to 43.5% in 2019. The vaccination rates of target groups were significantly higher than non-target group. Among the target groups, the elderly aged ≥65 years showed consistently high vaccination rates, while people with chronic diseases showed low vaccination coverage. In the last two years, the vaccination rates of children aged ≤12 years and pregnant women showed significant increases.

Among the sociodemographic factors, female gender, rural residency, and low educational level were significantly related to higher influenza vaccination coverage. Consistent findings have been reported in various countries [12,19–22]. Rural inhabitants and people with low educational level are more likely to work in fields that require more physical activities and have less social support, which puts them in greater need of preventive measures [23]. In addition, public health centers actively participating in NIP are mostly located in rural areas, providing easier access to rural inhabitants in South Korea [24]. Yet some of the factors have small actual differences but obtained statistical significance by large sample size, such as the difference between 10–12 years vs. ≥13 years of education in Table 3, which requires cautious interpretation.

In our previous study that evaluated the trends in influenza vaccination from 2005 to 2014, we showed that the vaccination rates of the elderly ≥65 years and children <5 years were significantly higher than other target groups [12]. These two groups were receiving financial aids under NIP: for the elderly, influenza vaccinations were free of charge; for children, most vaccines other than influenza were granted for free. Moreover, the positive correlation of high household income with high vaccination rates was selectively absent in these two groups [12]. Based on these findings, we have suggested that free vaccination policies successfully removed the financial barrier and improved vaccination rates.

The current study revealed a significant increase in vaccination rates in two target groups: children aged ≤12 years and pregnant women. Again, we suggest the correlation with recent alterations of policies. As NIP expanded its financial coverage in recent years, free vaccinations have been provided to children aged 6 months to 5 years since 2017, for children aged ≤12 years since 2018, and for pregnant women since 2019 [8]. As in Fig 1, the vaccination coverage of children aged ≤12 years had remained consistent then started to rise in 2017—along with the introduction of free vaccinations. For pregnant women, although the trend shows fluctuations due to the small sample size, the vaccination rates were slowly increasing before the policy alteration in 2019, which may arise from educational campaigns and increased efforts in obstetric clinics. A serial survey study in South Korea has reported that influenza vaccination coverage of pregnant women steadily increased (4.0% in 2006; 42.1% in 2011; 59.6% in 2018) along with the increase in the proportion of patients who received their doctors’ recommendation (2.8% in 2006; 36.8% in 2011; 49.7% in 2018) [25]. However, there was an additional abrupt increase after the initiation of financial aids in 2019, which was substantial enough to be statistically significant despite the low sample size and hindered statistical power. Therefore, we suggest that financial aids were the main cause of the recent improvements in vaccination coverage in both groups.

The impact of free vaccination policies has been repeatedly reported in the literature. A Cochrane review has evaluated its effect with two randomized controlled trials, which compared free vaccination vouchers versus mere invitations for vaccination that the participants should have paid. Free vaccination vouchers led to higher vaccination rates, with a pooled odds ratio of 2.36 (95% CI 1.98–2.82; p-value <0.001) [26]. Dyda et al. performed a systematic review on influenza and pneumococcal vaccination rates in Australian adults from 1990 to 2015, which revealed the coverage of both vaccines was significantly higher following the introduction of universal funding (for influenza, pre and post-funding, 61.3% vs 74.8%, p<0.001) [27]. Howard et al. also reported large increases in vaccination rates for children <5 years in 2018 that coincided with the introduction of funded vaccines in Australia (OR 4.75, 95% CI 4.57–4.79) [28].

South Korea adds a good example of national immunization programs and free vaccination policies. In 2014, only 59% of WHO members had national immunization policies [29]. Not so many countries are providing free influenza vaccinations to high-risk populations, which includes Australia, New Zealand, and the United Kingdom. Influenza vaccination rates are similarly high in these countries: comparing in the elderly ≥65 years in 2019, Korea marked 85.8%, UK 72.4%, and New Zealand 62.0% (Australia unavailable) [11].

The target group that presented with the lowest vaccination rates was the people with chronic diseases. Complex determinants drive vaccination trends—for example, individual knowledge, vaccine confidence, financial access, general health-system strength, and political commitment [30]. In survey studies, the factors associated with higher vaccination coverage in this group were the awareness of the potential severity of influenza, the receipt of healthcare recommendations, and high income [31,32]. Several strategies have been proposed to address each factor: awareness and educational campaigns, alerts or tracking systems in the electronic medical charts, and the expansion of financial aids [33]. Based on our observations in other target groups, we suggest that financial coverage should be expanded to people with chronic diseases. It has been reported that people with chronic diseases do not tend to engage themselves in active health behaviors such as abstinence, smoking cessation, or exercise [34]. This tendency is especially well reported for people with liver diseases, who showed the lowest vaccination coverage in this study [35]. As it is somewhat doubtable that this population would respond favorably to campaigns or recommendations, we suggest that financial incentives should take the primary role. Other interventions should be added with the expectation of synergistic effects, which includes educational campaigns or alert systems in electronic charts to encourage recommendation from healthcare personnels.

We have estimated the actual number of target populations in S1 Fig. In 2018, 13 million individuals could receive free influenza vaccination in South Korea [13]. The expansion of free vaccinations can be considered in two directions: age and disease. Age-wise legislation is easily approachable: we can expand it to the adolescents aged 13–18 years or adults aged 50–64 years who are also designated as high-risk populations. However, as in S2 Fig, the number of these populations adds up to 16 million, requiring double the budget. Instead, we can expand it to people with chronic diseases. The number of individuals aged 19–64 with chronic diseases is estimated to be 11 million. Moreover, individuals with higher risks can be recognized by evaluating the severity of diseases. To focus the budget on the population with higher risks of morbidity and mortality, we suggest the expansion of financial coverage to people with chronic diseases in order of severity.

The COVID-19 pandemic can play a role. The expansion of financial coverage has been tackled not only because of budget but also because of the lacking capacity of manufacturing facilities for vaccines. However, these facilities were largely installed recently for the rapid supply of COVID-19 vaccines. After sufficient COVID-19 vaccination for the general population is established, we could repurpose these facilities for seasonal influenza, which could result in significantly enhanced vaccine supply. Moreover, the importance of vaccination for people with underlying diseases is emphasized more than ever, with the widespread understanding of the efficacy of vaccinations in reducing the morbidity and mortality of COVID-19. With prompt awareness campaigns, this notion could smoothly be transitioned to influenza to help us overcome the long-lasted avoidance of vaccinations by people with chronic diseases for the fear of side effects.

Furthermore, it is concerning that there is no real-time monitoring system for influenza vaccination rates in South Korea. Like many other countries, Korea operates an active sentinel surveillance system in which influenza-like illnesses (ILI), as well as influenza-associated mortality and virus polymerase chain reaction results, are reported from selected medical centers and laboratories [36]. The results are released in weekly reports to assess the prevalence and impact of seasonal influenza of the time. However, influenza vaccination rates are not included in this surveillance system. Real-time monitoring of vaccination rates enables us to identify and correct issues regarding vaccine supply, access and communication with healthcare facilities [37]. In addition, there is no global monitoring system for influenza vaccination coverage [38]. We suggest that the real-time surveillance system for vaccination coverage be developed on a national and global scale to distribute the vaccines more efficiently to meet the demands of the time [30].

In this study, we discussed the factors that contribute to increased vaccination coverage, but it is controversial whether further increasing vaccination rates from this point will lead to reduced influenza incidence or not. Some data support its effect: sentinel surveillance data of South Korea has shown that ILI in children aged 1–6 years has been decreasing since the introduction of free vaccinations in 2017 [39]. A single-center study also reported that the number of children aged ≤5 years diagnosed with influenza has decreased between 2014 and 2018 [40]. In contrast, the opposite reports also exist: an age-period-cohort analysis with National Health Insurance Research Database from 2009 to 2018 has revealed that the crude incidence rate of influenza has been continuously rising in every age-specific cohort including the children despite the constantly increasing vaccination rates [41]. The authors suggest the role of antigenic mismatch between the vaccine and circulating viruses, particularly because trivalent influenza vaccines have been selected for free vaccination under NIP, while quadrivalent vaccines should be administered at individuals’ expense. In addition, yearly variation of the epidemic pattern, circulating influenza strain and its characteristics, as well as the efficacy of vaccines can also contribute to ILI. In conclusion, it is undeniable that the receipt of vaccination is essential in preventing influenza, but vaccination coverage alone may not be sufficient in diminishing the socioeconomic burden. Various factors should be considered in concert to enhance actual public health benefits, such as the precise expectation of circulating influenza strains, proper production and distribution of vaccines, maintenance of personal hygiene, and early diagnosis and management of influenza.

This study is not without limits. First, as a cross-sectional study, we do not provide direct comparisons between interventions. We interpreted the changes in vaccination rates in light of financial aids under NIP and called for its expansion. However, it remains no more than a hypothesis that should be challenged by prospective studies, yet the design and performance are difficult in the real-world setting. Second, the vaccination status was self-reported by surveys. It holds potential risks of recall bias. Third, KNHANES does not include the institutionalized population who are also designated as high-risk of influenza. Also, other high-risk groups such as healthcare workers, people living with high-risk individuals, and people with immunodeficiency could not be identified with KNHANES. Lastly, the surveys failed to assess the reasons for vaccination non-compliance, which would be interesting to include in further studies.

## Conclusions

We evaluated the trends in influenza vaccination rates in South Korea from 2010 to 2019 with the data from a nationwide survey—KNHANES. The discrepancy between target groups corresponded to their financial coverage under NIP. The recent introduction of free vaccination to children aged ≤12 years and pregnant women was followed by significant increases in vaccination rates in these groups. We suggest that free vaccination policy is one of the most effective strategies in enhancing vaccination coverage, and we call for its expansion to other target groups, especially people with chronic diseases.

## Supporting information

S1 Fig The estimation of the actual number of target populations. (TIF)

S2 Fig The estimation of the actual number of potential target populations. (TIF)

S1 Table The comparison of high-risk groups designated by CDC and KDCA. (DOCX)

S1 File Data set. (DTA)

S2 File Code book. (DOCX)
