# Supplementary figures and images for: The impact of free vaccination policies under the Korean Influenza National Immunization Program: Trends in influenza vaccination rates in South Korea from 2010 to 2019
Source: PLoS One. 2022 Jan 20;17(1):e0262594. doi: 10.1371/journal.pone.0262594 (PMC8775253; doi:10.1371/journal.pone.0262594)

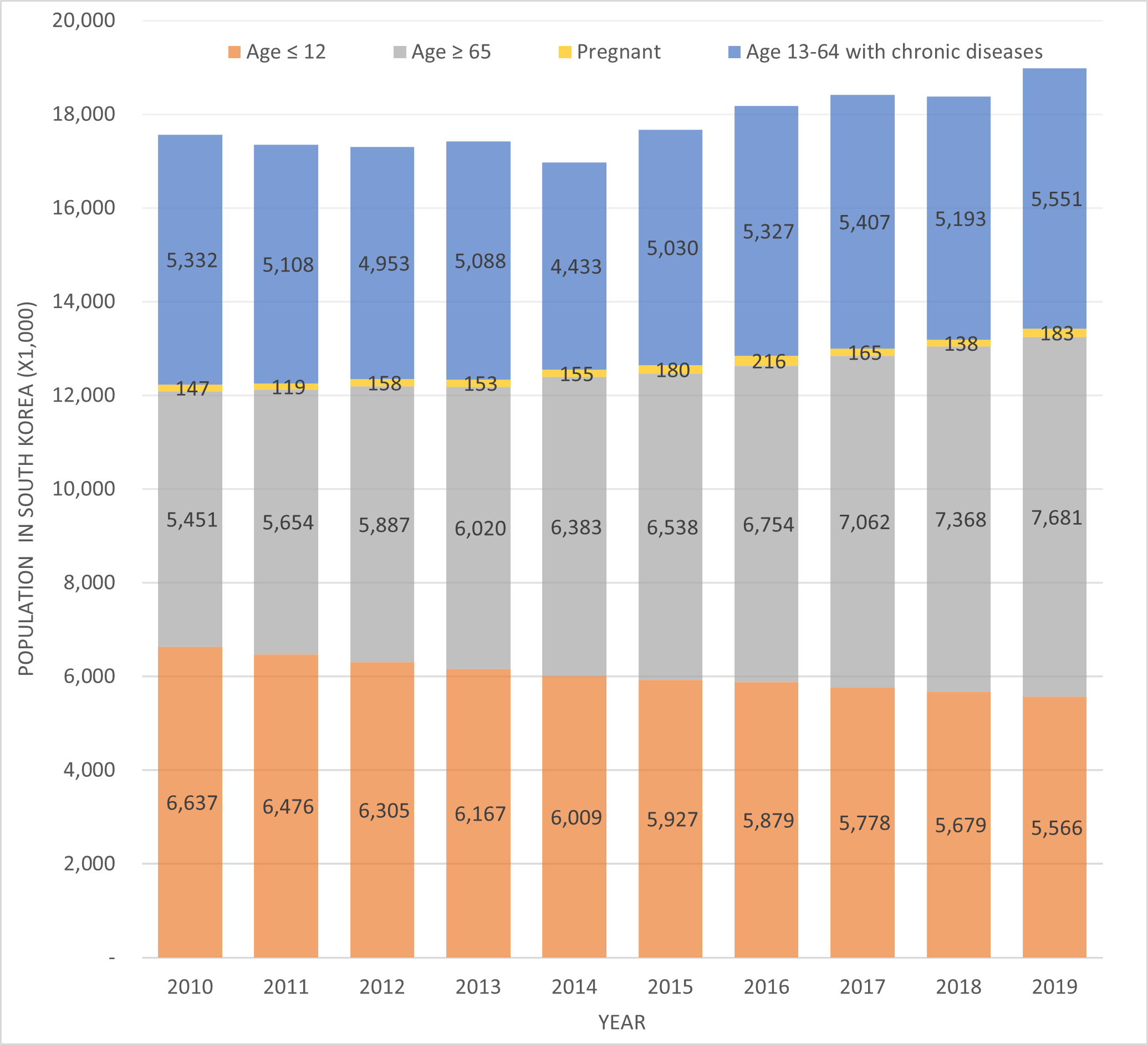

Supplement: S1 Fig — (TIF) [file pone.0262594.s001.tif]

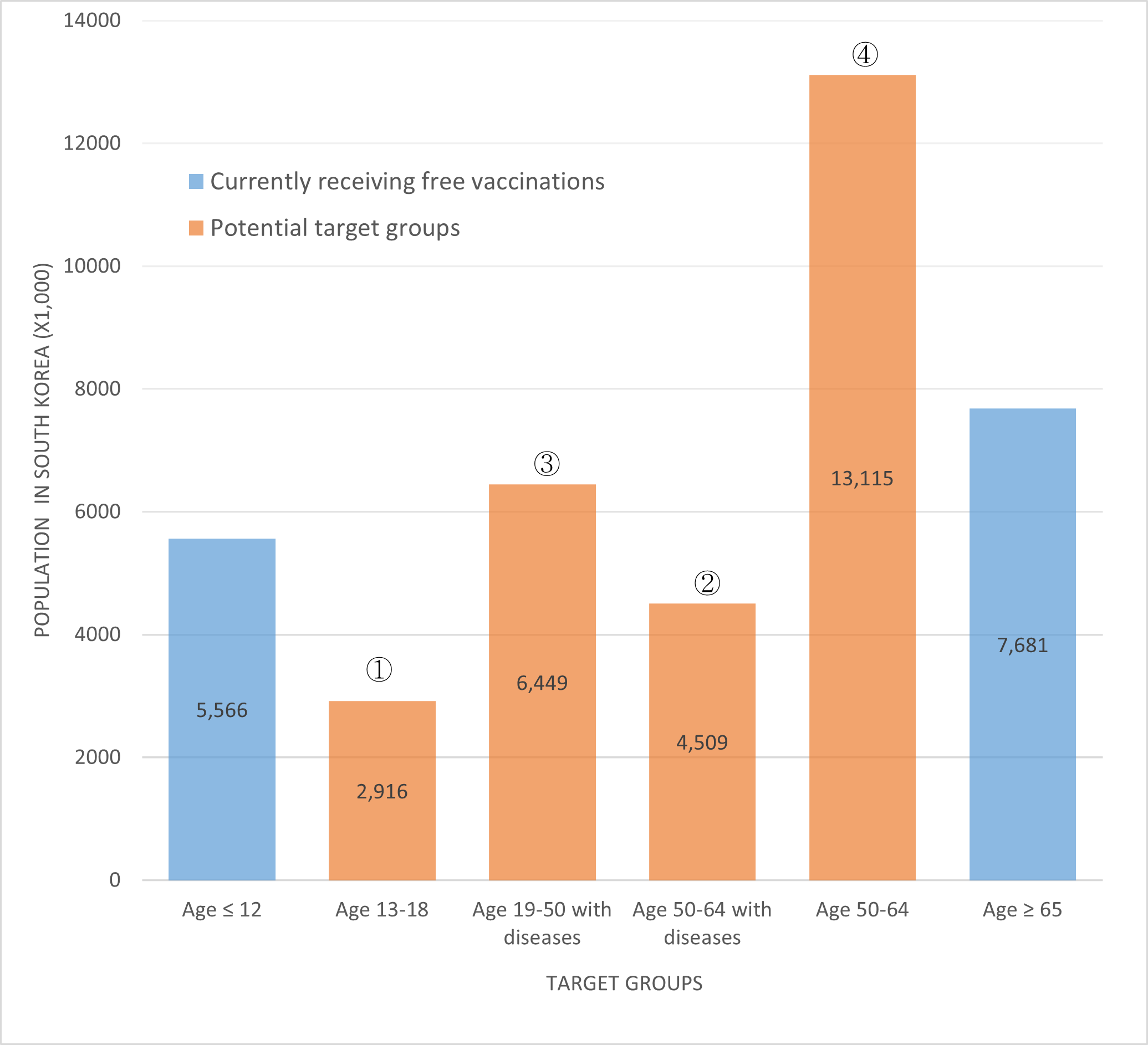

Supplement: S2 Fig — (TIF) [file pone.0262594.s002.tif]
