# Supplementary material for: The impact of free vaccination policies under the Korean Influenza National Immunization Program: Trends in influenza vaccination rates in South Korea from 2010 to 2019
Source: PLoS One. 2022 Jan 20;17(1):e0262594. doi: 10.1371/journal.pone.0262594 (PMC8775253; doi:10.1371/journal.pone.0262594)
Supplement: S1 Table — (DOCX) [file pone.0262594.s003.docx]

| **CDC** | **KDCA** |
| --- | --- |
| - Children aged 6 – 59 months | - Children aged 6 – 59 months^b^ |
| - All persons aged ≥50 years | - The elderly aged ≥65 years^b^ - Adults aged 50 – 64 years |
| - Women who are or will be pregnant during the influenza season | - Pregnant women^b^ |
| - Adults and children who have chronic diseases^a^ | - People with chronic diseases^a^ |
| - Persons who are immuno-compromised due to any cause | - Persons who are immuno-compromised due to any cause |
| - Children and adolescents (aged 6 months through 18 years) who are receiving aspirin- or salicylate-containing medications and who might be at risk for experiencing Reye syndrome after influenza virus infection | - Children and adolescents (aged 60 months through 18 years) who are receiving aspirin |
| - Residents of nursing homes and other long-term care facilities | - Individuals institutionalized for chronic diseases^a^ |
| - Persons who live with or care for persons at higher risk for influenza-related complications: healthcare personnel and household contacts | - Healthcare personnel - Individuals living with infants aged <6 months, the elderly aged ≥65 years, pregnant women, and people with chronic diseases |
| - American Indians/Alaska Natives - Persons who are extremely obese (body mass index ≥40 for adults) | - Persons who live in close and prolonged proximity: adolescents aged 60 months – 18 years |

^a^ Chronic pulmonary (including asthma), cardiovascular (excluding isolated hypertension), renal, hepatic, neurologic, hematologic, or metabolic disorders (including diabetes mellitus)

^b^ Individuals receiving free vaccinations under Korean National Immunization Program for Influenza

*CDC: Center for Disease Control and Prevention; KDCA: Korea Disease Control and Prevention Agency
