# Supplementary material for: The impact of free vaccination policies under the Korean Influenza National Immunization Program: Trends in influenza vaccination rates in South Korea from 2010 to 2019
Source: PLoS One. 2022 Jan 20;17(1):e0262594. doi: 10.1371/journal.pone.0262594 (PMC8775253; doi:10.1371/journal.pone.0262594)
Supplement: S2 File — (DOCX) [file pone.0262594.s005.docx]

CODE BOOK

| Name of Variables | Labels |
| --- | --- |
| year | Survey year |
| age | Age(year) |
| sex | Gender |
| town_t | Residual area |
| ainc | Home income per month |
| edu | Education level |
| bh9_11 | Self-reported influenza vaccination status |
| he_prg | current pregnant |
| di3_dg | stroke |
| di4_dg | coronary heart disease |
| di5_dg | myocardial infarction |
| di6_dg | angina |
| dj2_dg | tuberculosis |
| dj4_dg | bronchial asthma |
| dk4_dg | liver cirrhosis |
| dk8_dg | chronic viral hepatitis, B |
| dk9_dg | chronic viral hepatitis, C |
| ca | malignancies |
